# Ethnobotanical studies of fodder grass resources for ruminant animals, based on the traditional knowledge of indigenous communities in Central Punjab Pakistan

**DOI:** 10.1186/s13002-017-0184-5

**Published:** 2017-10-04

**Authors:** Nidaa Harun, Abdul Shakoor Chaudhry, Shabnum Shaheen, Kifayat Ullah, Farah Khan

**Affiliations:** 1 Department of Botany, Lahore College for Women University, Lahore, 54000 Pakistan; 2 School of Natural and Environmental Sciences, Newcastle University, Newcastle upon Tyne, NE1 7RU UK; 3 Department of Biosciences, COMSATS Institute of Information Technology, Islamabad, 45320 Pakistan

**Keywords:** Traditional knowledge, Central Punjab, Fodder grasses, Ruminants

## Abstract

**Background:**

Traditional knowledge of indigenous plants is pivotal in developing strategies to feed livestock sustainably in low input systems. Likewise, in Pakistan the indigenous people of Central Punjab have been using their regional grasses as a ruminant fodder for centuries. This study evaluated the indigenous traditional knowledge to ascertain the value of various fodder grasses to optimise their use to feed livestock in Central Punjab.

**Methods:**

The snowball technique was employed to identify key informants who had relevant knowledge about different grasses in the study area. Semi-structured questionnaires, face-to-face interviews and site visits were used for describing the fodder grasses. The data were then analysed by using relative frequency citation and pairwise comparison methods to determine the order of priority among the listed fodder grasses. Furthermore, SPSS 22 software was used for descriptive statistics and interpretation of associations among studied parameters. Microsoft Excel was used to present data as % values and graphs.

**Results:**

Overall, 53 grasses were described with ethnobotanical information regarding their uses for fodder, ethnoveterinary and other purposes. All these grasses belonged to the family Poaceae where the subfamily Panicoideae had the maximum number of 30 grasses. We categorized these grasses into high (A), medium (B) and low priority (C) groups where the group A grasses were reported as not only the most abundant but also the most palatable forages to all ruminants. Their higher demand was reflected by the feeding systems of both ad libitum grazing and feeding after cutting and mixing with other feeds. The study also revealed 37 previously unreported ethnoveterinary uses of these grasses.

**Conclusions:**

The results have reinforced the value of conserving ethnobotanical knowledge, being poorly documented previously, in developing strategies to feed livestock. It indicated the preferred fodder grasses as well as the possible reasons of their preference. The reported data need to be validated for nutritional and health benefits. This information could help the smallholder farmers in association with regional governments to propagate suitable fodder grasses for their use in sustainable livestock feeding to produce safe and healthy food for indigenous communities.

## Background

The agriculture and livestock industry are playing a predominant role in Pakistan’s economy. Around 43.5% individuals are linked with this industry with its 21% contribution in Gross Domestic Product (GDP). In this sector the most protuberant role has been made by the Punjab province in comparison to all other provinces of Pakistan [1].

Geographically Punjab is subdivided into south, north, west and central regions [2]. Amongst all the regions of Punjab the Central region is primarily involved in the production of milk and meat from ruminants. Although this area is blessed with diversified fodders (trees, shrubs, herbs and grasses), grasses are conventionally the most common and reliable fodder source for ruminant animals. The indigenous people prefer to use grass as a fodder because grasses are observed to be more palatable than shrubby fodders by ruminant animals [3–6]. Moreover the grasses have massive growth abilities around different seasons and these are conveniently more accessible. Therefore, this study aimed to provide comprehensive information on the traditionally used fodder grasses of Central Punjab Pakistan.

Indigenous communities which have been involved in livestock handling possess a significant knowledge about potential forage resources [7]. Many countries (e.g. India, Ethiopia, Nigeria, Mexico, China and Uganda) around the world understand the worth of this traditional knowledge and therefore they had documented this classic data about fodder plants from various ethnic groups [7–10]. Even in Pakistan multiple ethnobotanical studies have been carried out in different cities of Central Punjab but the previous studies were more focussed on the ethnomedicinal values rather than the fodder significance of indigenous plants. Additionally, these studies seemed to be mostly engaged with fodder trees, herbs or shrubs and not with grasses [11–15]. However grasses are one of most promising fodder resources of this region. While a few ethnobotanical studies involving grasses were conducted in some regions of Pakistan, their main focus was to evaluate the significance of those grasses for human health [16]. Inadequate records about the traditionally used fodder grasses of this region indicated the vulnerability of particular traditional knowledge to being vanished and overlooked Therefore it is crucial to manuscript this traditional knowledge about the preference for fodder grasses by the rural communities of Central Punjab, Pakistan.

This ethno botanical survey based study not only aimed to describe many traditionally used fodder grasses but also to set out an order of priority on the basis of their usage for different ruminant animals. The study also evaluated the relative abundance, medicinal worth, delectableness, and feeding systems of these grasses for ruminants.

## Methods

### Study area

**Fig. 1:**
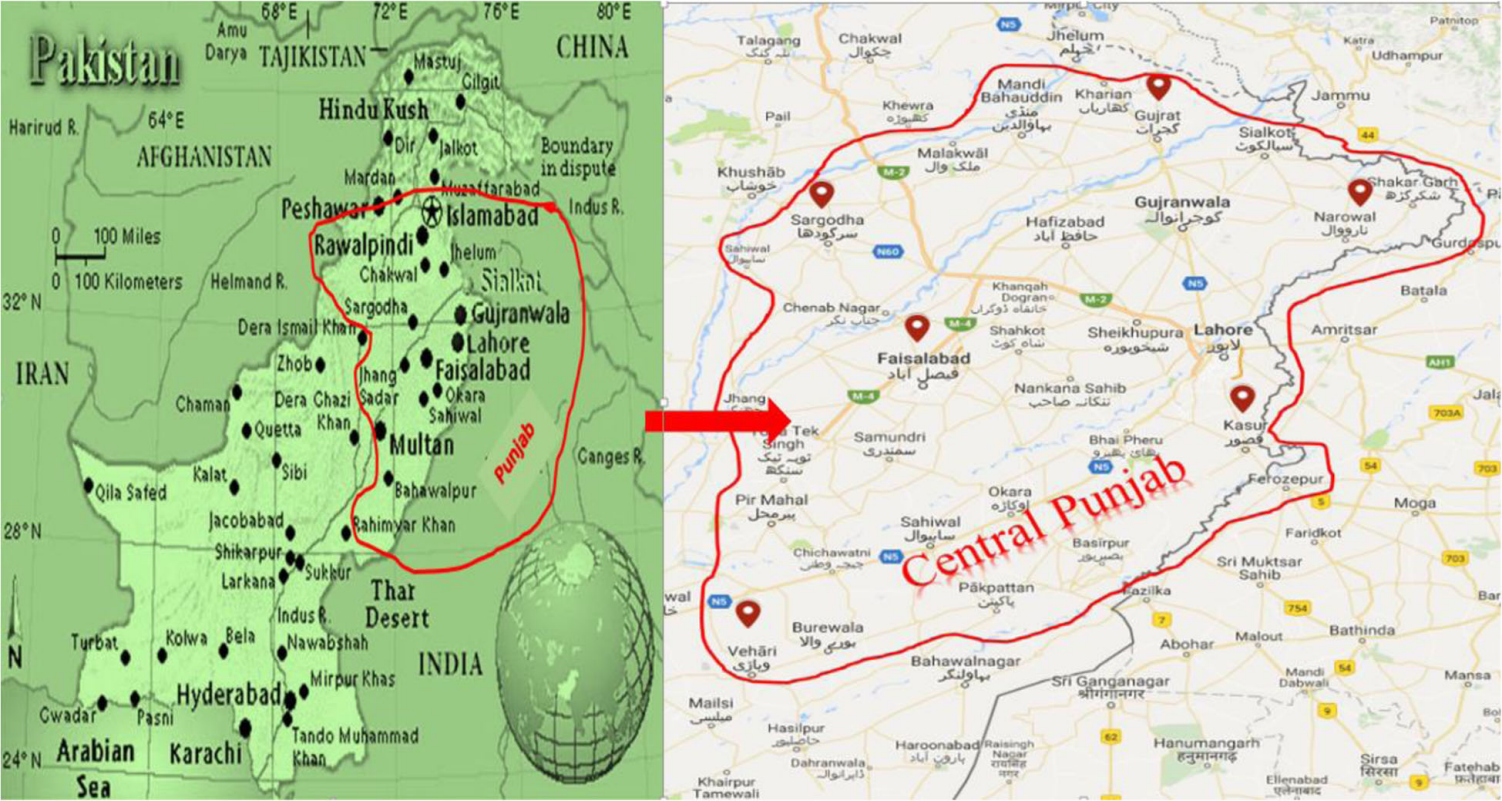
Map of study area showing all major cities of Central Punjab, Pakistan and encircled cities covered by this study

The southern boundary of River Jhelum down to River Sutlej surrounds the planes of Central Punjab. This region is comprised of 19 districts which are grouped into 3 agro-ecological zones. Among these 3 zones 6 representative cities i.e. Kasur, Faisalabad, Vehari (Northern irrigated zone), Sargodha (Sandy deserts zone), Gujrat, and Narowaal (Barani zone) were selected. As the northern irrigated zone is the largest zone, maximum numbers of 3 targeted areas were selected from it. These areas are not only the main producers of ruminant milk and meat but also these are distantly apart from each other which helped the collection of diversified ethnobotanical data from this region. In these districts, the remote rural areas were actually targeted due to their reliance on conventional fodder grasses as a feed for raising their ruminant animals (Fig. 1).

### Ethnobotanical survey and data collection

The primary goal of this survey was to collect ethnobotanical information about fodder grasses from local ruminant caretakers. Before collecting the data, formal ethical permission was obtained from the chairperson of local government and individual informants of selected study areas. The data were collected between the months of March 2014 to February 2015 from remote and less developed villages of targeted cities. A total of 137 informants were chosen by employing snowball sampling technique. The informants included male and female village leaders, shepherds, ruminant caretakers who worked in indigenous farms and some senior domestic animal caretakers in each of the selected areas (Table 1). Group discussions and individual ethnobotanical semi-structured interviewing techniques were used for data collection [17, 18]. The questionnaires were constructed in English. However, for ease in communicating with the local people during interviews and group discussions their indigenous language (different dialects of Punjabi) was used and the answers were translated back to English. The questionnaire included the following questions: (1) Which fodder grasses are most likely to be fed to their ruminant animals? (2) What is the palatability of their chosen grasses? (3) Which part did the animals consume? (4) What are their feeding mode, were they free grazing alone or supplemented or offered as cut grass mixed with other feeds? (5) Do the listed fodder grasses have any ethno veterinary use? (6) What are their other indigenous uses apart from the fodder and ethno veterinary uses?

**Table 1:** Demography of informants of this study area

Type of Informants	Young aged	Middle aged	Seniors	Total
	25–35 years	36–50 years	51–65 years	
Local shepherds (Female)	2	5	0	7
Local shepherds (Male)	13	23	7	43
Farmed Ruminant care takers (Female)	2	4	3	9
Farmed Ruminant care takers ((Male)	9	11	3	23
Domestic Ruminant care takers (Female)	11	15	8	34
Domestic Ruminant care takers (Male)	6	12	3	21
Total informants	43	70	24	137

### Fodder grass sampling and authentication

For the identification and collection of fodder grass samples, several site visits were made with some knowledgeable indigenous people. They helped the surveyor in identification and collection of particular fodder grass from its habitat. The details of each specimen i.e. date of collection; habitat, local names and flowering periods were also recorded during each site visit.

After their collection, each fodder grass sample was identified by comparing their morphological characters with already available grass specimens in the herbariums of Lahore College for Women University, Lahore and the Quaid i Azam University, Islamabad. Along these two herbaria, online available plant databases like flora of Pakistan (http://www.efloras.org/index.aspx), flora of India (https://sites.google.com/site/efloraofindia/) and some other grass flora identification keys [19, 20] were also consulted for their identification and authentication. Afterward the voucher numbers were allotted to all specimens, which were then submitted to the Botany Herbarium of Lahore College for Women University (LCWU).

### Estimation of relative abundance

The most commonly used method of visual assessment was employed for measuring the relative abundance of ethnobotanically enlisted grass species in study area [21]. In this method number of plots randomly selected in study area and the presence of each listed species were counted and recorded. Afterwards percentage relative abundance was calculated by using the following formula;

$$ \mathrm{Relative}\  \mathrm{abundance}\  \mathrm{of}\  \mathrm{species}=\frac{\mathrm{Total}\  \mathrm{percentage}\  \mathrm{Cover}\  \mathrm{of}\  \mathrm{species}\  \mathrm{in}\ \mathrm{all}\ \mathrm{plots}}{\mathrm{Number}\  \mathrm{of}\  \mathrm{plots}\  \mathrm{estimated}}\times 100 $$

The species were then grouped into different categories i.e. Abundant, Common, Frequent, Occasional and Rare (ACFOR) by using relevant scales of abundance (Table 2).

**Table 2:** Abundance categories and scale of reported grasses

Abundance scale	Abundance categories	Coverage of grass species
+	Rare (R)	<5%
1	Occasional (O)	5–20%
2	Frequent (F)	20–50%
3	Common (C)	50–90%
4	Abundant (A)	90–100%

### Data analysis

All the recorded data values were tabulated by using Microsoft excel 2013. Two data analysis methods i.e., Relative Frequency of Citation and Pairwise comparison method were applied to find out the priority order of their grass utilization as described below:

- Relative frequency of citation (RFC)

This tool helped us to set up the priority order among the listed fodder grasses. Its value depended upon the numbers of respondents that had mentioned a particular grass species as a good fodder indicating its significance. The RFC was estimated by using the following eq. [22].

$$ \mathrm{RFC}=\mathrm{FC}/\mathrm{N}\left(0<\mathrm{RFC}<1\right) $$

where

FC = number of respondents who stated that particular grass species as a good fodder, N = total number of respondents included in study

b) Pairwise comparison method (PC)

In combination to RFC another data analysis tool called PC method was also employed to establish a priority order among listed fodder grasses [23]. In this method a comparative matchup chart (Table 3) was constructed between different fodder grasses and then each informant was asked to vote their preferable fodder grass among those. Each species got 1 point if the respondents preferred it over the other fodder grass. The half point was allotted to each of them if they were ranked equal by the respondents. Finally, all points were added for each grass species to predict their priority order of utilization.

c) Cluster analysis & descriptive Statistics

**Table 3:** Template of comparative matchup chart used for pairwise comparison for different grasses

Fodder grasses	Species A	Species B	Species C	Species D	Species E	Total votes	Rank
Species A	…………						
Species B		……………					
Species C			………….				
Species D				………….			
Species E					…………		

For making groups of high and low priority fodder grasses, Hierarchical Cluster Analysis (Squared Euclidean distance method) in the SPSS 22 software was applied to the RFC values. Moreover, descriptive statistical analysis (frequency and cross tabulation) was also employed to find out the association between different parameters of the survey.

d) Graphical illustrations

Microsoft Excel was used to convert selected data items into different types of graphical illustrations.

## Results and discussions

### Demography of study area

**Fig. 2:**
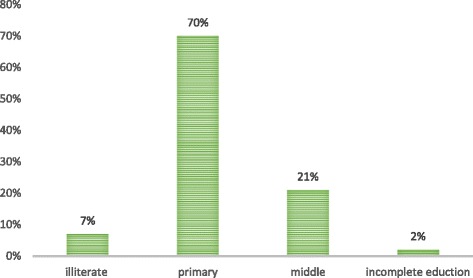
Summary of education levels of informants

The informants of this study were divided into 3 major age groups i.e. 25–35 years, 36–50 years and 51–65 years. The maximum number of informants was local shepherds (41%) because they were the key users of these fodder grasses. It was also observed that in this category most informants were males (84%) and the rest were females (16%). Similarly, in farms there were more men (72%) than women (28%). The less number of females as shepherds and farmers showed the cultural pattern of the study area where females were not expected to work in an outdoor environment in this region. Therefore, much higher (60%) number of females was recorded for the category of domestic animal caretakers (Table 1). Regarding their education level, most respondents had completed either 5 years of primary (70%) or 8 years of middle (21%) level education and a few with no education (7%) alongside 2% with incomplete education (Fig. 2). The animal care takers working in farms were different from the domestic animal care takers in terms of their education. Most of those had 8 years of education with additional training in animal handling and hygiene control measures. It was interesting to note that almost all the informers who were relying on wild grasses as a fodder for their animals, were financially not very sound. Therefore, one of the possible reason for them to utilize these grasses, could be that these grasses were a cost free fodder resource for them to use as a feed for their ruminant animals.

### Taxonomic diversity of fodder grasses

**Fig. 3:**
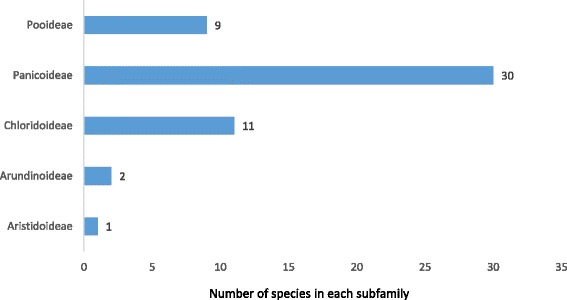
Number of fodder grass species in each subfamily

This study revealed that ruminants of Central Punjab Pakistan were fed on a diversified range of wild grasses. As expected, the grass types and their availability did vary between and within the 3 agro-ecological zones of Central Punjab. However, no distinction for this variation was made between these zones when assessing the data for this comprehensive report on 53 ethnobotanical fodder grasses of Central Punjab, Pakistan (Table 4). It appeared during the taxonomic identification process that all of these documented fodder grass species were members of the family Poaceae which is well known for its fodder significance. The value of Poaceae family as fodders is recognised by various ethnobotanical studies from various regions such as those in Africa, India China and even in the lesser Himalayan and Thal dessert of Pakistan [7, 24–28] These ethno botanically listed species belonged to 39 genera which had links with 8 different tribes and 5 subfamilies i.e. Aristidoideae (Aristideae), Arundinoideae (Arundineae), Panicoideae (Paniceae, Andropogoneae), Chloridoideae (Eragrostideae, Chlorideae) and Pooideae (Aveneae, Bromea). Among these subfamilies, Panicoideae was ranked as the top with 30 fodder members and subfamily Aristidoideae attained the least position because it had only 1 member grass being was used as a fodder (Fig. 3). Similar fodder value of subfamily Panicoideae has been well supported by the literature [29].

**Table 4:** Ethnobotanical descriptions, uses, abundance; focal persons count (FC) and relative frequency citation (RFC) of 53 fodder grasses

Subfamily	Voucher no.	Binomial name	Local name	Palatable to	Fodder part	Feeding method	Ethno veterinary uses	Other uses	RA	FC(n)	RFC
Pooideae	LCWU-0360	*Agrostis gigantea* Roth.	Lamba gaah	Ca, Sh	Leaves	FG, MF	used against allergic reactions	NR	C	85	0.62
	LCWU-0385	*Avena sativa* L.	Jai	Ca, Bu, Sh, Go	Aerial	FG, MF	Detoxifier	Part of human food	F	51	0.372
	LCWU-0364	*Bromus japonicus* Thunb.	Joukai	Go, Sh	Aerial	FG, MF	Treat constipation	Crop cover during harsh winters	C	82	0.599
	LCWU-0386	*Dactylis glomerata* L.	Gadu	Go, Sh	Leaves	FG	Diuretic	NR	O	33	0.241
	LCWU-0387	*Lolium temulentum* Linn.	Cockle	Sh, Go	Leaves	FG	Nervous disorders	NR	O	33	0.241
	LCWU-0377	*Phalaris minor* Retz.	Dumbi sitti	Sh, Go	Aerial	FG	Extract helpful to cure animal cough	keep mouse at bay from wheat storage areas	A	76	0.555
	LCWU-0389	*Poa annua* L.	Poa	Ca	Whole	FG	Remove debris from wounded area	NR	F	51	0.372
	LCWU-0388	*Poa infirma* Kunth.	Wakh	Ca, Go	Whole	FG	NR	NR	F	49	0.358
	LCWU-0391	*Polypogon monspeliensis* (L.) Desf.	Malhar	Sh, Go	Aerial	FG	Infusions used to normalize the increased heart palpitations	NR	F	47	0.343
Arundinoideae	LCWU-0361	*Arundo donax* L	Nara bans, Nal	Ca, Bu	Leaves	FG, MF	Diuretic, Antiseptic	Dried plant parts used as fuel and shelters. Stems also used to make flutes	A	74	0.54
	LCWU-0390	*Phragmites australis* (Cav.) Trin. ex Steud.	Dila	Ca, Sh	Leaves	MF	Digestive disorders	Used in construction of adobe houses. The mature and dried stems also used in making of musical instruments.	O	31	0.226
Aristidoideae	LCWU-0391	*Aristida adscensionis* Linn.	Lumb Gaah	Ca, Sh	Aerial	FG	Controls itching	Revegetation, stabilizes sand dunes	F	43	0.314
Chloridoideae	LCWU-039	*Acrachne racemosa* (Heyne ex Roth) Ohwi	Chinki	Ca	Whole	FG	NR	Grain used by people in food scarcity	O	29	0.212
	LCWU-0369	*Cynodon dactylon* (Linn.) Pers.	Khabbal, Tala, Chaber	Ca, Bu, Sh, Go	Whole	FG, MF	Paste of leaves controls dysentery and anti-inflammatory to wounded areas of animal’s body	NR	A	95	0.693
	LCWU-0370	*Dactyloctenium aegyptium* (L.) Wild.	Koora, Madanah	Ca, Sh	Whole	FG	Used to reduce after birth abdominal pains	NR	A	78	0.569
	LCWU-0371	*Desmostachya bipinnata* (L.) Stapf	Kusa, Dab	Ca, Bu	Aerial	FG, MF	Digestive disorders, Dysentery	Used as roof covers and in making of brooms	A	76	0.555
	LCWU-0373	*Eleusine indica* (L.) Gaertn.	Chezi	Ca	Aerial	FG, MF	Cure digestive disorders	Used in making ropes and mats	A	91	0.664
	LCWU-0392	*Enneapogon persicus* Boiss.	Jiu	Ca	Whole	FG	NR	NR	F	46	0.336
	LCWU-0395	*Eragrostis japonica* (Thunb.) Trin.	Panghas	Ca, Bu, Sh, Go	Whole	FG, MF	NR	NR	F	48	0.35
	LCWU-0374	*Eragrostis minor* Host.	Choti ghas	Ca, Bu, Sh, Go	Whole	FG, MF	Digestive disorders	seeds used as food in food scarcity times	A	90	0.657
	LCWU-0393	*Eragrostis pilosa* (L.) P. Beauv.	Nika sanwak	Ca, Bu, Sh, Go,	Whole	FG	Help to cure contusion	NR	O	52	0.38
	LCWU-0398	*Leptochloa panicea* (Retz.) Ohwi	Paja	Ca	Whole	FG	NR	Basketry material	R	33	0.241
	LCWU-0399	*Tetrapogon villosus* Desf.	Sager	Ca	Aerial	FG	NR	NR	R	39	0.285
Panicoideae	LCWU-0400	*Apluda mutica* L.	Tachuli	Ca	Aerial	FG	Disinfectant, Digestive disorders	used for thatching with combination of other grass materials	R	30	0.219
	LCWU-0362	*Bothriochloa bladhii* (Retz.) S.T. Blake	Palvan	Ca, Bu, Sh, Go	Aerial	FG, MF	Improves digestion	helps in re vegetation	A	91	0.664
	LCWU-0363	*Brachiaria ramosa* (Linn.) Stapf	Sawari, Jhanda	Ca	Whole	FG	Leaves work as antiseptic	NR	F	51	0.372
	LCWU-0396	*Brachiaria reptans* (Linn.) Gardner & Hubbard	Hausa	Ca	Whole	FG	Leaves juice helps to cure anaemia, also used as laxatives	Sometime seeds used as food	C	84	0.613
	LCWU-0397	*Cenchrus biflorus* Roxb.	Bhurat	Ca, Bu	Aerial	FG	Diuretic	NR	F	52	0.38
	LCWU-0365	*Cenchrus ciliaris* L.	Dhaman	Ca, Bu	Aerial	FG, MF	Diuretic	NR	A	88	0.642
	LCWU-0366	*Cenchrus pennisetiformis* Steud.	Bara Dhaman	Ca, Bu	Whole	FG	NR	Herbicidal	C	80	0.584
	LCWU-0401	*Cenchrus setiger* Vahl.	Kala dhaman	Ca	Aerial	FG	Antiseptic	NR	R	35	0.255
	LCWU-0367	*Chrysopogon aucheri* (Boiss.) Stapf	Khar, Chorkanda	Ca, Bu, Sh, Go	Aerial	FG, MF	Digestive disorders	NR	A	78	0.569
	LCWU-0368	*Chrysopogon zizanioides* (L.) Roberty	Vetiver, Khuss	Ca	Leaves	FG, MF	Antiseptic, anti-inflammatory	Used in rehabilitation of land, also for making barriers around territories	A	78	0.569
	LCWU-0402	*Cymbopogon jwarancusa (*Jones.) Schult	Khavi, Kathori	Ca, Bu, Sh, Go	Whole	FG	Diuretic and improve fertility in bull	Extract used as mosquito repellent	O	28	0.204
	LCWU-0372	*Dichanthium annulatum* (Forssk.) Stapf	Palwan, Murgha	Ca, Bu, Sh, Go	Whole	FG, MF	Digestive disorders	Considered herbicidal	A	91	0.664
	LCWU-0403	*Digitaria ciliaris* (Retz.) Koeler	Shamokha	Sh, Go	Whole	FG	NR	NR	O	50	0.365
	LCWU-0407	*Digitaria longiflora* (Retz.) Pers.	Deeta	Sh, Go	Whole	FG	NR	NR	O	45	0.328
	LCWU-0408	*Echinochloa colona* (L.) Link	Jungli chowol	Ca, Bu, Sh, Go	Whole	FG	Digestive disorders	Stems are used for weaving mats and sometime used as food.	F	52	0.38
	LCWU-0404	*Echinochloa crus-galli* (L.) P. Beauv.	Sanwak	Ca	Whole	FG	Digestive disorders	Seldom used for reclamation of alkaline soils	O	48	0.35
	LCWU-0375	*Heteropogon contortus* (L.) P Beauv. Ex. Roem & Schult.	Pili, Butto jara	Ca	Aerial	FG, MF	Digestive disorders	handicrafts and thatching	C	73	0.533
	LCWU-0376	*Imperata cylindrica* (L.) Raeuschel	Siru	Ca, Bu, Sh, Go	Whole	FG	Fumigant for Piles	biological pesticide	A	83	0.606
	LCWU-0405	*Ottochloa compressa* (Forssk.) Hilu	Chimbar, Phalwan	Ca	Aerial	FG	NR	NR	R	35	0.255
	LCWU-0406	*Panicum antidotale* Retz.	Gharam	Ca	Whole	FG	Disinfectant	NR	O	61	0.445
	LCWU-0409	*Paspalidium distichum* L.	Knot grass	Ca	Whole	FG	NR	NR	O	53	0.387
	LCWU-0501	*Pennisetum orientale* Rich.	Haathi ghaa	Ca	Whole	FG	Oral infections	NR	O	60	0.438
	LCWU-0378	*Saccharum bengalense* Retz.	Kana, Sarkand A	Ca, Bu, Sh, Go	Leaves	FG, MF	Leaves used to treat oral problems of ruminants	Dried plants used as fuel, thatching and making of writing pens	C	78	0.569
	LCWU-0379	*Saccharum spontaneum* L	Kaa	Ca	Leaves	MF, FG	Root help to relieve in inflammation and urinary problems	Used as paper pulp as well as in making of ropes, baskets and brooms.	C	76	0.555
	LCWU-0381	*Setaria pumila* (Poir) Roem. & Schult.	Ban kangni	Ca	Aerial	FG, MF	Oral infections	Used to tie knot bundles of grain together	A	85	0.62
	LCWU-0500	*Setaria verticillata* (L.) P. Beauv.	Barchittas	Sh, Go	Leaves	FG	Flatulence problem	Used in making of baskets	O	27	0.197
	LCWU-0380	*Setaria viridis* (L.) P. Beauv.	Kangni	Ca, Bu, Sh, Go	Whole	FG, MF	Seeds used to treat bruises and also effective as diuretic.	NR	A	91	0.664
	LCWU-0382	*Sorghum bicolor* (L.) Moench	Jowar	Ca, Bu, Sh, Go	Aerial	FG, MF	Help to cure wounds, anaemia and constipation	Part of human food and also used to make sweet syrups	C	78	0.569
	LCWU-0383	*Sorghum halepense* (L.) Pers.	Baru	Ca, Bu, Sh, Go	Aerial	FG, MF	Decotion of root used to reduce the swelling of mammary glands	As fuel	A	86	0.628
	LCWU-0384	*Zea mays* L.	Makai	Ca, Bu, Sh, Go	Leaves	FG, MF	Treat sores and skin problems	Kernel and oil used as food for humans	A	87	0.635

*NR* Not Reported, *FG* Free grazing, *MF* Mixed with feed, *Ca* Cattle, *Bu* Buffalo, *Sh* Sheep, *Go* Goat, *RA* Relative abundance, *A* Abundant, *C* Common, *F* Frequent, *O* Occasional, *R* Rare

### Prioritizing fodder grasses on the basis of RFC and PC

**Fig. 4:**
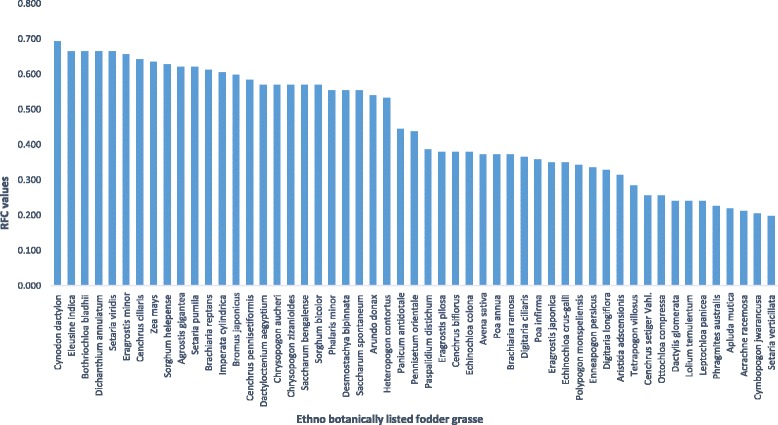
Prioritizing of fodder grasses on the bases of RFC

**Fig. 5:**
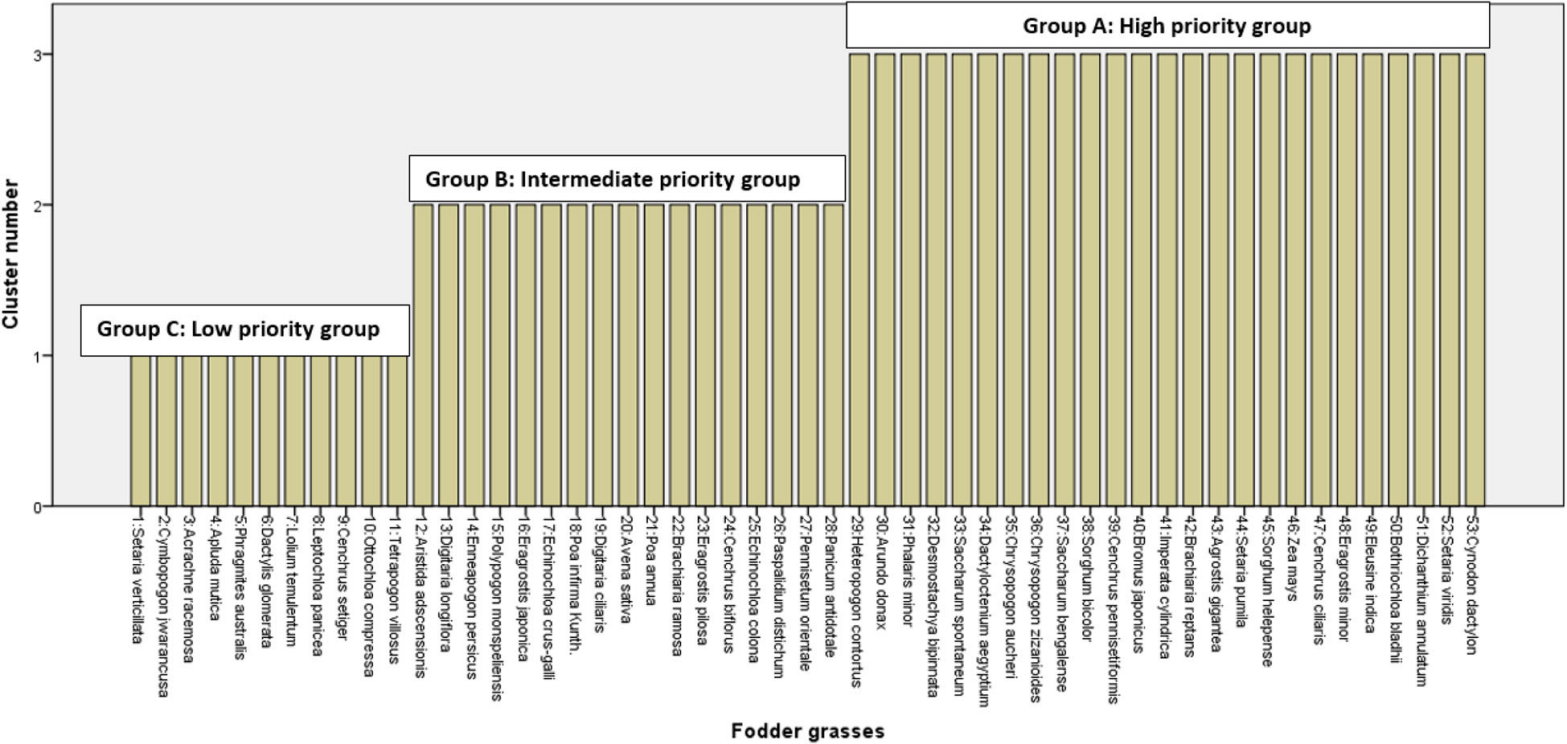
Cluster analysis for grouping of ethno botanically used fodder grasses

The priority determined by the RFC value of 0.693 to 0.197 showed the variable eminence of grasses as a fodder at different sites of study area (Fig. 4). For the sake of data management and comprehensive analysis of listed fodder grasses, they were clustered into high (A), medium (B) and low (C) priority groups on the basis of RFC (Fig. 5). However, when the groups were closely observed it was found that many of the grasses had the same RFC value even within the same group. So the question about their actual priority level was resolved by applying PC method and those fodder grasses which had similar RFC were reorganised in their priority order (Table 5).

**Table 5:** Pairwise comparison for fodder grasses having similar RFC

Fodder grasses	Total gained % points	Rank
GROUP A (RFC =0.664)		
*Bothiochloa Bladhi*	87.5	1st
*Dicanthium annulatum*	85.5	2nd
*Setaria Viridis*	84.5	3rd
*Eleusine indica*	84	4th
GROUP B (RFC =0.554)		
*Setaria pumila*	87	1st
*Agrostis gigantea*	79	2nd
GROUP B (RFC = 0.518)		
*Dactyloctenium aegyptium*	89.2	1st
*Chrysopogon aucheri*	88	2nd
*Chrysopogon zizanioides*	86	3rd
*Saccharum bengalense*	80	4th
*Sorghum bicolor*	79.6	5th
GROUP C (RFC = 0.474)		
*Phalaris minor*	92.6	1st
*Saccharum spontaneum*	90.6	2nd
*Desmostachya bipinnata*	87.3	3rd
GROUP D (RFC = 0.379)		
*Echinochloa colona*	59	1st
*Cenchrus biflorus*	55	2nd
*Eragrostis pilosa*	54	3rd
GROUP D (RFC = 0.372)		
*Brachiaria ramosa*	36	1st
*Avena sativa*	35.3	2nd
*Poa annua*	34.6	3rd
GROUP D (RFC = 0.35)		
*Echinochloa crus-galli*	31.3	1st
*Eragrostis japonica*	30	2nd
GROUP E (RFCs = 0.255)		
*Cenchrus setiger*	27	1st
*Ottochloa compressa*	29	2nd
GROUP E (RFC = 0.24)		
*Dactylis glomerata*	30	1st
*Lolium temulentum*	29.3	2nd
*Leptochloa panicea*	27.3	3rd

**Fig. 6:**
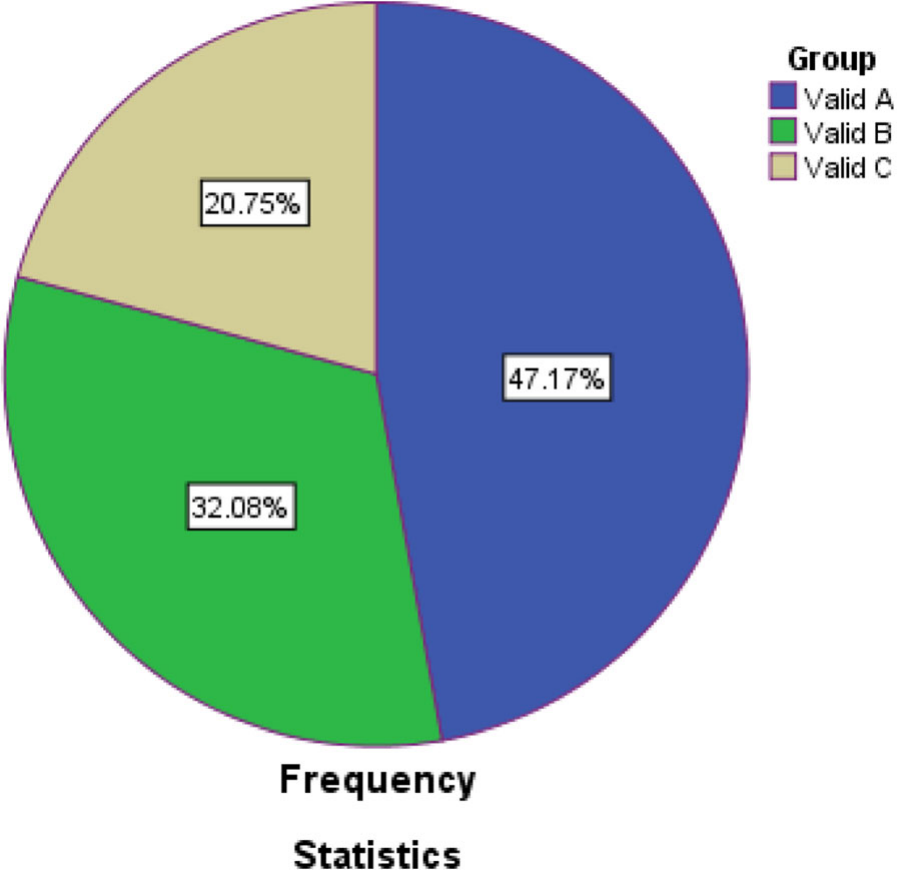
Percentage of species in each group

**Fig. 7:**
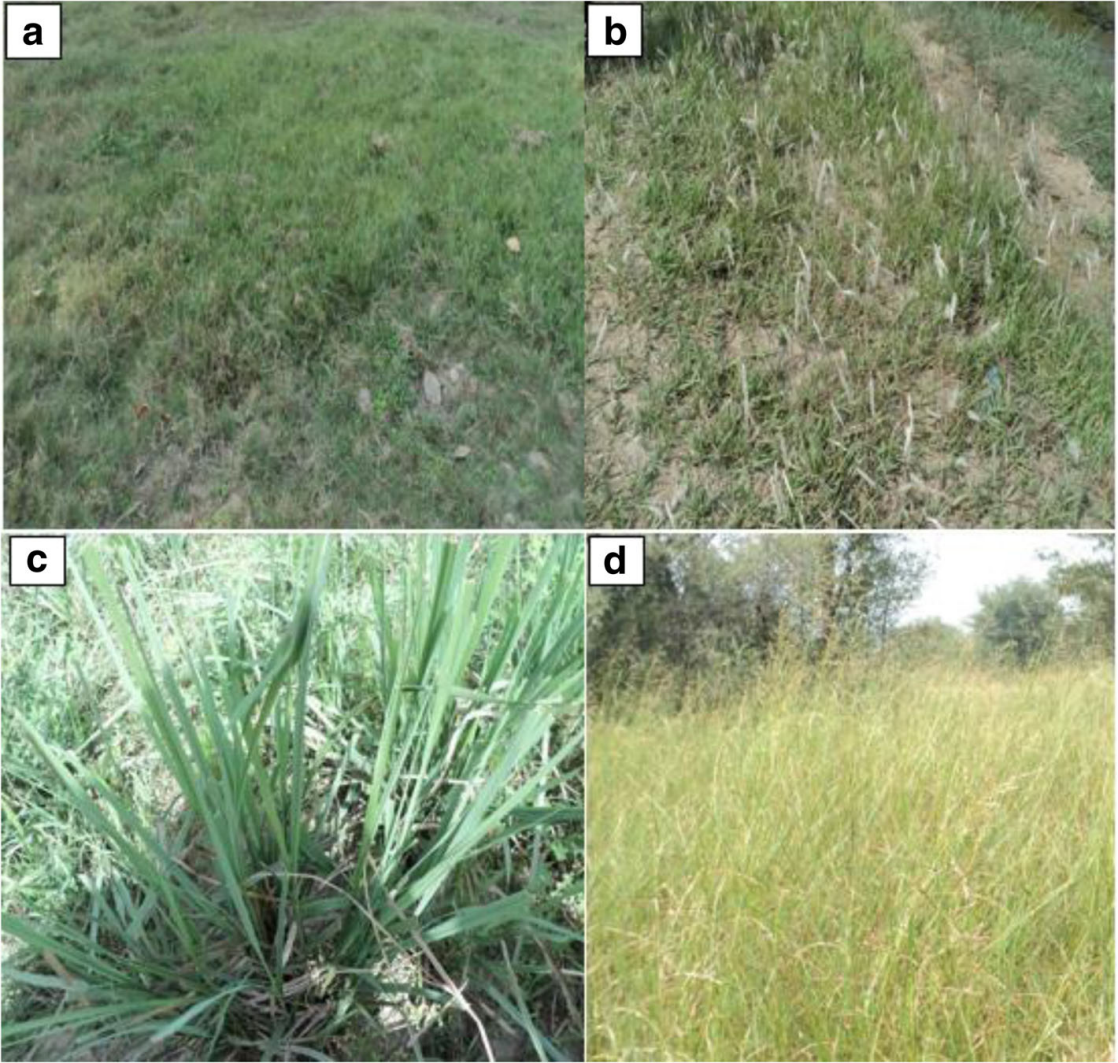
Examples of some fields with selected members of high priority fodder grasses group e.g. (**a**) *Cynodon dactylon,* (**b**) *Imperata cylindrical,* (**c**) *Saccharum spontaneum,* (**d**) *Sorghum halepense*

The RFC of group A (high priority) ranged from 0.693 to 0.533 and this group comprised of 25 grasses (Fig. 5). However, the group B (medium priority) ranged from 0.445–0.314 with 17 species and group C was extended from 0.285–0.197 RFC, with 11 species of fodder grasses (Figs. 5 and 6). The higher RFC of top most priority groups (A) depicted that these fodder grasses were probably more dominant in the study area and indigenous people had more familiarity with this group of grasses [30]. So it can be said that all those fodder grass species belonging to high priority group A (*n* = 25) were the most likely and most preferably utilized fodder grasses by the indigenous communities. These fodder grasses were preferred because of their availability, palatability, ability to satisfy animal hunger, ease in availability, positive effects on milk production and shelf life during a dry season (Fig. 7 a-d). Despite the fact that these fodder grasses were valued by local people as a ‘quality’ fodder, it is essentially required to assess the nutritional potential of these fodder grasses for the sustainability of healthy and efficient livestock industry.

### Palatability, part used and feeding methods of listed fodder grasses

**Fig. 8:**
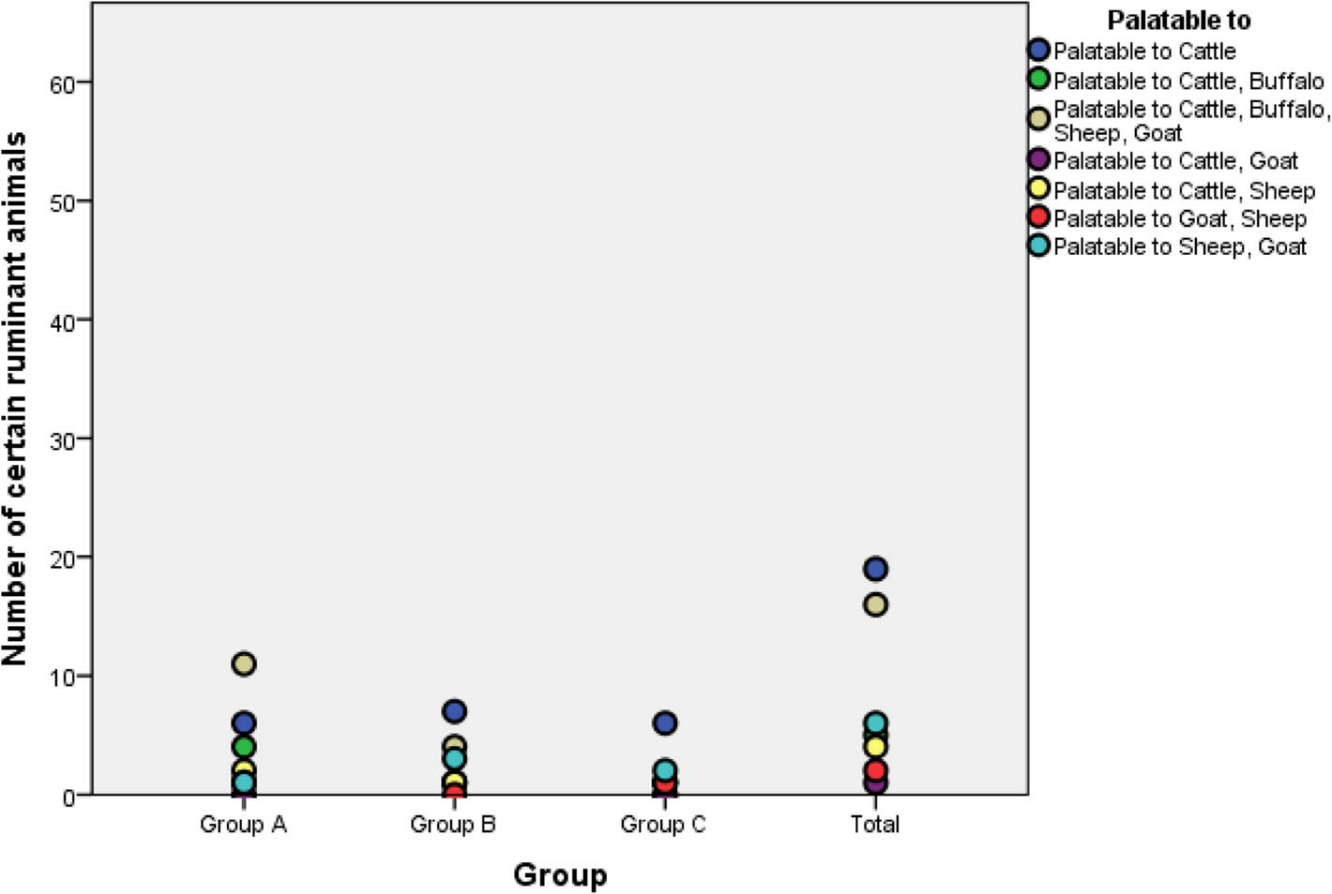
Association between palatability and priority groups of grasses through cross tabulated method

Palatability is the dietary characteristics which can elicit a specific response from an animal [31, 32]. Statistical analysis of palatability frequency analysis showed that all of these fodder grasses were most commonly palatable for cattle i.e. cumulatively 77% (Table 6). However, the cross tabulated results showed that grasses of group A were palatable to all categories of locally found ruminants i.e. cattle, buffalo, sheep and goat (Fig. 8). The high palatability of group A members for all types of ruminants indicated their more wide acceptance and significance as highly preferable fodders.

**Table 6:** Descriptive statistics: frequency analysis for palatability, parts used for eating and feeding methods and relative abundance of fodder grasses

Studied parameters	Frequency	Valid percent	Cumulative percent
Cattle	19	35.8	35.8
Cattle, Buffalo	5	9.4	45.3
Cattle, Buffalo, Sheep, Goat	16	30.2	75.5
Cattle, Goat	1	1.9	77.4
Cattle, Sheep	4	7.5	84.9
Goat, Sheep	2	3.8	88.7
Sheep, Goat	6	11.3	100.0
Total	53	100.0	
Fodder part			
Aerial	19	35.8	35.8
Leaves	10	18.9	54.7
Whole	24	45.3	100.0
Total	53	100.0	
Feeding methods			
Free grazing	30	56.6	56.6
Free grazing, mixed with feed	21	39.6	96.2
mixed with feed	2	3.8	100.0
Total	53	100.0	
Relative abundance			
Abundant	17	32.1	32.1
Common	8	15.1	47.2
Frequent	10	18.9	66.0
Occasional	13	24.5	90.6
Rare	5	9.4	100.0
Total	53	100.0

**Fig. 9:**
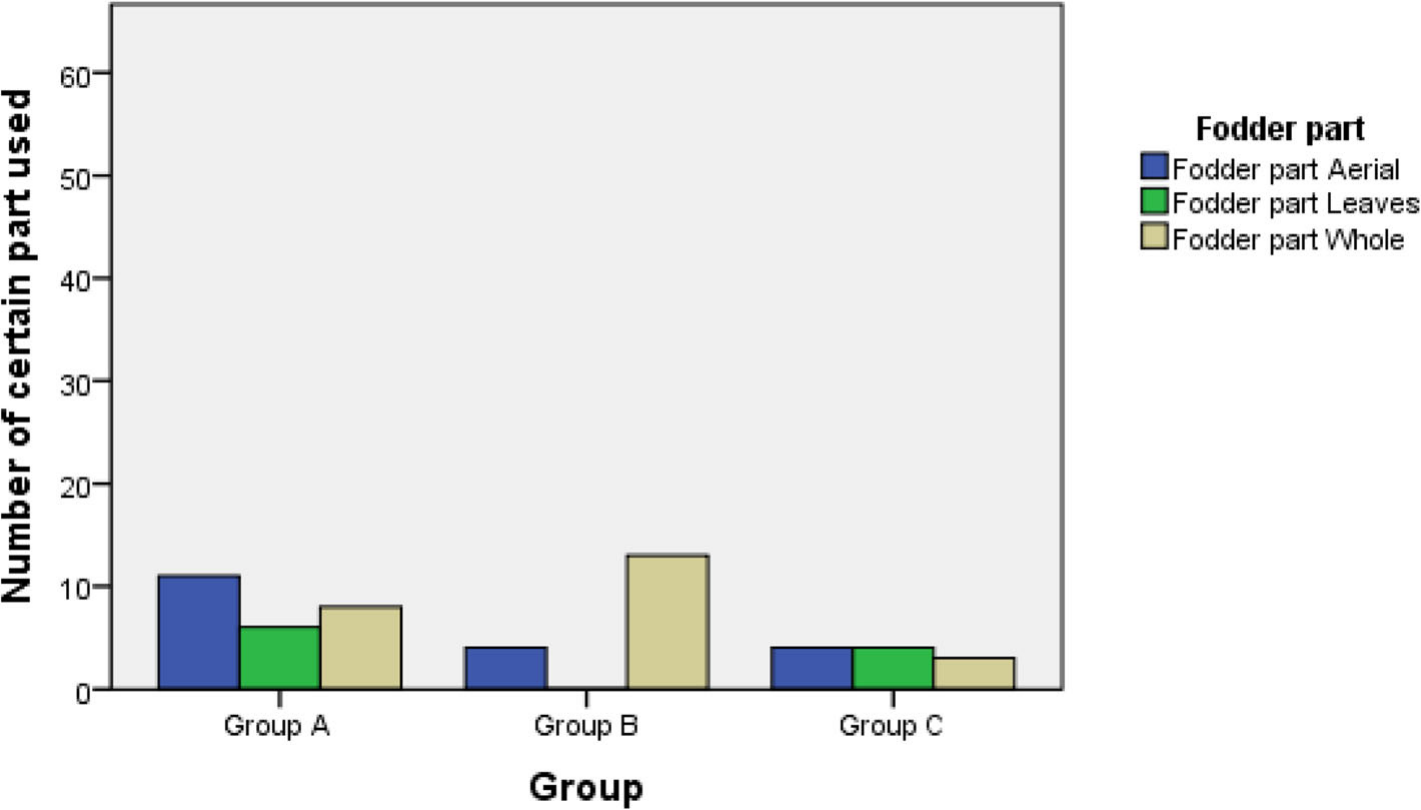
Association between the usage of fodder part and priority groups of grasses through cross tabulated method

It appeared that most of these fodder grasses were used as a whole plant i.e. 45%, followed by the use of aerial parts 35.8% and leaves 18.9% (Table 6). The maximum reported percentage for the whole plant use was probably due to the fact that majority of these grasses were small in height, herbaceous in nature with non woody fibrous and shallow roots which were easily pulled out of soil by the animals. However cross tabulation between priority groups and fodder parts indicated that the group A grasses were mostly eaten by their aerial parts. This is because 9out of 25 grasses (i.e., *Sorghum halepense, Desmostachya bipinnata, Sacchrum spontaneum, Saccharum bengalense, Chrysopogon zizanioides, Arundo donax, Sorghum bicolor, Agrostis gigantean, Zea mays*) of this group were above the 200 cm or 400 cm in heights and these grew well in vigour and density that animal didn’t need to pull up the whole plant to satisfy its hunger (Fig. 9).

**Fig. 10:**
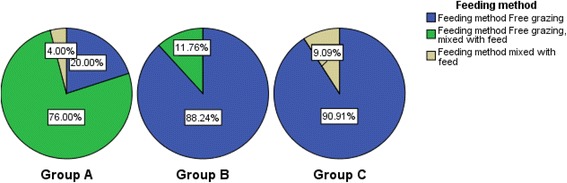
Association between feeding method and priority groups of grasses through cross tabulated method

The current results revealed that overall all the ethno botanically listed grasses were most frequently fed to ruminant animals through ad libitum grazing (cumulatively 96.2%) (Table 6). Ruminants were probably comfortable with ad libitum grazing due to the fact that they have the natural capability to either avoid the ingestion or utilization of the ingested toxic plants [33, 34]. The provision of grass also plays a valuable role in the production of good quality meat from cattle. Indeed the beef from grass fed animals would be rich in polyunsaturated fatty acids with lower cholesterol content than the beef from animal fed high grain diet [35].It is interesting to report that the members of group A were fed by either grazing or cut and mixed with other type of feeds (Fig. 10). This can be attributed to the high demand and speedy regrowth of these high priority grasses of group A in different regions of this study.

### Relative abundance of listed fodder grasses

**Fig. 11:**
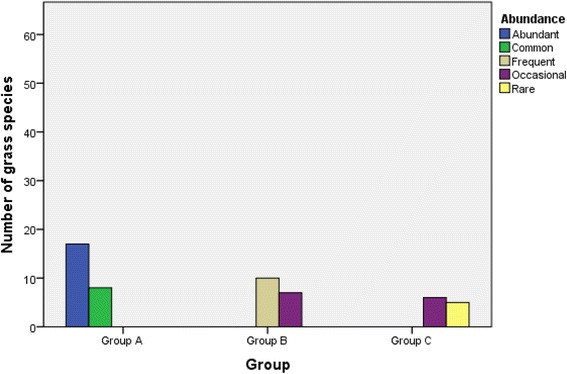
Association between abundance and priority groups of grasses through cross tabulated method

The results of relative abundance showed that most of the listed fodder grasses were abundant in study area i.e. 32.1%, while least number of fodder grasses was found to be rare (9.4%) (Table 6). Also an interesting relevance was observed between abundance and priority levels (Fig. 11). The fodder grasses of group A were mostly observed as abundant (17) and common (8) however the medium priority level fodder grasses (B) were recorded as frequent (10) or occasional (7). The lower priority level fodder grasses (C) were customarily in the occasional (6) or rare (5) category. This revealed that the abundance of fodder grasses directly affects their priority of utilization. The grasses which were more abundant in this study area were more preferably used as compared to the others which were less abundant.

### Ethno veterinary and other indigenous uses of listed fodder grasses

Since ancient times the human beings are using plant resources for medicinal purpose for not only themselves but also their livestock [36]. This use of plants for animal health care is termed as ethnoveterinary which was evolved alongside animal domestication [37] .The use of these indigenous plants to address multiple health issues of their livestock facilitated animal keepers to decrease the unaffordable cost of certain veterinary medicines [38]. This traditional ethnoveterinary practice is playing a compelling role in maintaining animal production around the globe where rural communities mainly rely on livestock for their livelihood [39–45].

Among all ethno botanically listed fodder grasses, 43 grasses were found with ethno veterinary significance (Table 4). This data showed that local people not only feed their animals on these grasses but also use them to treat the mild health disorders of ruminant animals. Grasses like *Bromus japonicus*, *Phragmites australis, Cynodon dactylon*, *Desmostachya bipinnata*, *Eleusine indica*, *Eragrostis minor* were used to treat the multiple digestive disorders like dysentery, constipation and flatulence problems. However, some served as antiseptics e.g. *Arundo donax*, *Brachiaria ramose*, *Sorghum bicolor*, *Panicum antidotale* and *Chrysopogon zizanioides*. The reported ethnoveterinary uses of all grasses were compared with other published data from different regions of Pakistan. Some studies stated similar ethnoveterinary uses for *Cynodon dactylon* [46, 47] while few documented different ethnoveterinary usages of same grasses like *Arundo donax, Saccharum spontaneum, Saccharum bengalense, Sorghum halepense* and *Zea mays* [47–51]. However rest of the fodder grasses never reported for their ethnoveterinary use either in Central Punjab or other regions of Pakistan.

Apart from their ethno veterinary value, 25 grasses were also reported for their other indigenous uses (Table 4). Like majority of them are utilized for thatching or making baskets and to cover the crops for protection from harsh weather such as cold winters (*Bromus japonicus*, *Arundo donax*, *Phragmites australis*, *Desmostachya bipinnata*, *Apluda mutica* and *Heteropogon contortus*). However, *Phalaris minor* and *Cymbopogon jwarancusa* were interestingly also used as mouse and mosquito repellents respectively.

## Conclusion

This ethnobotanical study is the first of its kind which not only describes 53 naturally grown indigenous fodder grasses of Central Punjab Pakistan, but also provides an inventory which manuscript their local names, most commonly used parts for fodder, diversity in palatability and feeding systems, abundance category and unreported ethnoveterinary uses as well. In addition this research also established 3 fodder grass categories based upon their utilization value. The data analysis highlighted the possible motives behind the greater acceptability ratio of high priority fodder grasses i.e. diversity in their palatability for major ruminant species (cattle, buffalo, goat, sheep), abundant availability in the study area and versatile feeding methods (ad libitum grazing or cut, carry and mixed with other feeds). This data enriched study is not only significant for the conservation of ethnobotanical knowledge but also it may help in facilitating the sustainable livestock feeding for ruminants. Subsequently, the information may play a major role in improving the livelihood of smallholder farmers.

Although these high priority grasses have been used for fodder purpose for centuries by indigenous people, the recorded traditional data were never verified on experimental grounds. So there is a chance that drastic climatic changes in the past centuries would have also altered the soil properties which could ultimately affect the nutritional and medicinal value of these grasses. It is quite possible that actual nutritional as well as pharmacological facts and figures would show entirely a different picture about these conventionally used fodder grasses. Hence, a blend of traditional and scientific knowledge is essentially required to produce worthwhile selection criterion for these fodder grasses. Moreover, if some of these grasses show promising nutritional and pharmacological values then the relevant policy makers should take necessary steps for their enhanced but economical cultivation by providing much needed support to the traditional farmers of the study regions. We believe that further support for the small holder farmers who are working hard despite the challenging environment is needed in this region enriched with traditional knowledge. Otherwise, this natural biodiversity of beneficial grasses could be damaged due to over and unregulated grazing risking the achievement of food security in these and other similar neglected regions of great significance.

## Abbreviations

A: Abundant
Bu: Buffalo
C: Common
Ca: Cattle
F: Frequent
FC: Focal person count
FG: Free grazing
Go: Goat
MF: Mixed with feed
N: total number of informants
NR: Not Reported
O: Occasional
PC: Pairwise comparison
R: Rare
RA: Relative abundance
RFC: Relative frequency citation
Sh: Sheep
SPSS: Statistical Package for the Social Sciences
